# Terahertz Science and Technology in Astronomy, Telecommunications, and Biophysics

**DOI:** 10.34133/research.0586

**Published:** 2025-01-22

**Authors:** Jing Li, Xianjin Deng, Yangmei Li, Jie Hu, Wei Miao, Changxing Lin, Jun Jiang, Shengcai Shi

**Affiliations:** ^1^ Purple Mountain Observatory, Chinese Academy of Sciences, Nanjing 210023, China.; ^2^ Microsystem and Terahertz Research Center, China Academy of Engineering Physics, Chengdu, Sichuan 610200, China.; ^3^ Institute of Electronic Engineering, China Academy of Engineering Physics, Mianyang, Sichuan 621999, China.; ^4^ Innovation Laboratory of Terahertz Biophysics, National Innovation Institute of Defense Technology, Beijing 100071, China.

## Abstract

This paper reviews recent developments and key advances in terahertz (THz) science, technology, and applications, focusing on 3 core areas: astronomy, telecommunications, and biophysics. In THz astronomy, it highlights major discoveries and ongoing projects, emphasizing the role of advanced superconducting technologies, including superconductor–insulator–superconductor (SIS) mixers, hot electron boundedness spectroscopy (HEB), transition-edge sensors (TESs), and kinetic inductance detectors (KIDs), while exploring prospects in the field. For THz telecommunication, it discusses progress in solid-state sources, new communication technologies operating within the THz band, and diverse modulation methods that enhance transmission capabilities. In THz biophysics, the focus shifts to the physical modulation of THz waves and their impact across biological systems, from whole organisms to cellular and molecular levels, emphasizing nonthermal effects and fundamental mechanisms. This review concludes with an analysis of the challenges and perspectives shaping the future of THz technology.

## Introduction

Terahertz (THz) radiation refers to electromagnetic waves ranging from 100 GHz to 10 THz, corresponding to wavelengths between 3 mm and 3 μm, sandwiched between the millimeter wave and the middle infrared band. It features a short wavelength, high penetration in the dust, and low photon energy with no ionization damage. It is also a band rich in characteristic fingerprints of molecular vibrations and rotations, with important applications in various fields [1]. However, the THz band is traditionally called the “terahertz gap” because generating and detecting the THz wave has been difficult. Expertise in electrical engineering and fields like optical engineering and materials science is required to address these challenges.

THz radiation has long been studied in radio astronomy for the cosmic microwave background (CMB), star and planet formation, and the interstellar medium (ISM). The relevant observation continues to revolutionize our understanding of the universe. In 1965, the discovery of CMB by Arno Penzias and Robert Wilson [2] started the campaign to verify its spectrum. The first detection of the *J* = 1-0 (115.271 GHz) of carbon monoxide (CO) [3] from the Orion nebula in 1970, the most important tracer for molecular H2, opened a new window for the observation of the cold universe, including mapping the molecular cloud, probing the star formation region [4], as well as investigation of the ISM [5]. After that, many large telescopes, including the James Clerk Maxwell Telescope (JCMT) in Hawaii, USA, the Nobeyama Radio Telescope in Nagano, Japan, the IRAM 30-m telescope in Granada, Spain, and the Delingha 13.7-m telescopes in Qinghai, China, were proposed and constructed to observe the code sky. The 1980s saw the development of superconducting detectors, especially the superconductor–insulator–superconductor (SIS) mixers, which feature quantum-limited noise performance and soon became the dominant heterodyne mixer for almost all the THz telescopes below 1 THz until today, including the Submillimeter Array (SMA) in Hawaii, USA, Northern Extended Millimeter Array (NOEMA) in French Alps, and Atacama Large Millimeter Array (ALMA) in Chajnantor, Chile. Many important discoveries, including direct imaging of the black hole in 2019 [6], have been achieved with SIS mixers. In the 1990s, the hot electron bolometer (HEB) mixer was proposed. Many important lines above 1.2 THz, such as CII (1.9 THz) and NII (1.46 THz), can be efficiently observed. Detecting helium hydride ion (HeH^+^) with SOFIA was a critical milestone in understanding early cosmic chemistry and the evolution of the universe [7].

**Fig. 1.**
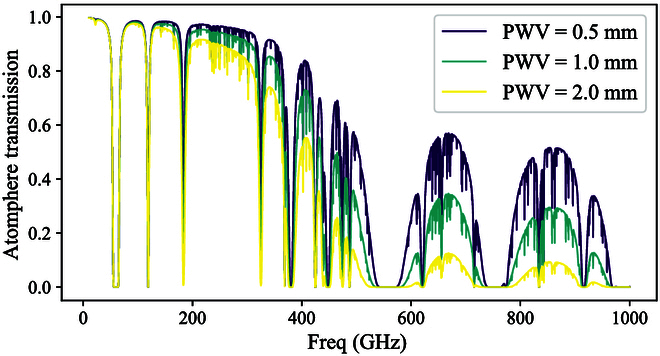
Simulated atmospherical transmission with PWV at an altitude of around 5,000 m.

With the advancement of the generation of the THz wave [8], THz applications in other fields like high-speed communications [9–11], atmospheric remote sensing [12,13], security imaging [14–17], and bio-sensing [18] have begun to be applied in the real world. In the last decade, there has been significant progress in THz telecommunications, particularly in terms of distance and data transmission speeds. This improvement has been largely driven by the demand for high-volume data transmission in next-generation telecommunication technologies [19–22]. However, THz communication remains strongly limited by atmospheric absorption, as illustrated in Fig. 1. These advancements have been made possible by breakthroughs in THz wave generation, especially through solid-state sources based on Schottky diodes [23–25]. As a result, THz telecommunication has become an area of intense competition among research groups worldwide.

In recent years, THz biophysics has emerged as a cutting-edge and deeply interdisciplinary field with encouraging progress, particularly in biodetection. For example, metamaterial-powered advanced THz biochemical sensing has exhibited high precision, robustness, and high sensitivity [18,26]. More notably, there is growing interest in studying how THz radiations explicitly affect complex neuron activities and biobehaviors [27]. It should be emphasized that for convenience, THz biophysicists refer to the much wider band of 0.5 to 100 THz (i.e., the traditional THz band to the mid-infrared band) as the generalized THz band [28,29] where the vibrational and rotational absorption spectra of biomolecules are located [30]. The challenge is that the thermal and nonthermal effects are hardly distinguishable due to heat energy accumulation via water absorption, which makes the physical mechanisms and laws intricate. But since water absorption windows exist [31], scientists start to focus on genuinely nonthermal and physical modulation of THz waves on the nervous systems, where water absorption is deliberately avoided to guarantee the THz energy reaching the target. Meanwhile, exploring how THz radiation acts on the hydrogen bond networks of water ubiquitous in life is very interesting, as it provides insights for interpreting THz bioeffects.

Due to the limited scale, this paper will focus on recent developments in THz sciences, technologies, and perspectives in astronomy, telecommunication, and biophysics. In THz astronomy, recent main discoveries and ongoing projects will be presented, highlighting significant advancements in this field. The chapter will also cover key superconducting technologies that have enabled these achievements, including SIS mixers, HEBs, transition edge sensors (TESs), and kinetic inductance detectors (KIDs). Additionally, the discussion will explore future perspectives and potential developments in THz astronomy. In THz telecommunication, recent key developments will be discussed, focusing on advancements in solid-state sources and the evolution of communication technologies operating in the THz band. It will also cover various modulation methods used in THz telecommunications, highlighting their roles and potential impact on the field. THz biophysics discusses the physical modulation of THz waves and biological impacts at different levels, from molecules and cells to whole organisms, and highlights the underlying mechanisms. The influential factors of physical modulation are analyzed, followed by perspectives on THz technologies applied in neuroscience.

## THz Astronomy

Observations in the THz band in astronomy are generally categorized into 3 main types: coherent detection, incoherent detection, and cosmic background radiation measurements. Coherent detection involves using phase-sensitive receivers, such as SIS or HEB mixers, which enable high spectral resolution and are well suited for observing molecular lines and other narrowband signals. Incoherent detection utilizes detectors such as bolometers, which measure the total power of incoming radiation without phase information, making them ideal for broadband observations of continuum emission. Lastly, cosmic background radiation observation measures the faint, almost uniform radiation field, often requiring susceptible instruments with stringent noise and stability requirements to capture these weak signals across the THz spectrum.

Astrophysical observations in the THz band can be done on the ground or in space. Earth’s atmosphere poses a significant challenge to THz observations because of its strong absorption of these signals, primarily caused by water vapor, as shown in Fig. 1, which calculates atmospheric transmission with different precipitable water vapors (PWVs) [32] at an altitude of around 5,000 m. This atmospheric absorption limits ground-based observations to a few narrow windows in the THz range, making site selection critical for maximizing observational efficiency. Ideal telescope sites are chosen based on their altitude, aridity, and atmospheric stability, which minimizes the presence of water vapor and improves the transmission of THz signals. Locations such as the Atacama Desert in Chile, Tibet in China, Mauna Kea in Hawaii, and the high-altitude regions of Antarctica offer some of the best conditions for ground-based THz astronomy. Space-borne observation has been pursued because it allows the whole spectrum in the THz band to be observed. Experiments that need to observe the full spectrum and that need to observe the lines absorbed by the atmosphere must be done above the atmosphere. Many space-based observations, such as COBE, WMAP, Planck, Herschel, and SOFIA, have been done to explore the universe.

In the THz band, 2 complementary approaches are used to observe the universe: single-dish observation and interferometry. Single-dish telescopes rely on a single large antenna to collect signals from the sky. This design provides a wide field of view and high sensitivity to diffuse and extended emissions, making them well suited for mapping large-scale structures. Notable examples of single-dish telescopes include the IRAM 30-m telescope, JCMT, and the Delingha 13.7-m telescope. However, the angular resolution of single-dish observations is limited by the size of the telescope. In contrast, interferometry uses multiple smaller antennas spread over a large area to combine their signals, achieving much higher angular resolution, equivalent to that of a telescope as large as the array’s maximum baseline. Prominent examples in the THz band include ALMA, NOEMA, and SMA. This approach is particularly effective for studying fine details in compact sources, such as the structures of protoplanetary disks or distant galaxies.

As high-resolution observations continue to be led by ALMA, the limitations of current single-dish submillimeter telescopes have become increasingly evident. These existing facilities are no longer able to keep up with the rapidly growing demands of modern astronomical research. Recognizing this challenge, astronomers now see the construction of large single-aperture submillimeter telescopes as a necessary step forward. These next-generation telescopes will offer a combination of high sensitivity, a wide field of view, and rapid sky-survey capabilities, addressing the needs of future astronomical observations. To meet these goals, Japan, Europe, and the United States are focusing on the development of groundbreaking facilities such as the Large Submillimeter Telescope (LST) [33] and the Atacama Large Aperture Submillimeter Telescope (AtLAST) [34], driven by cutting-edge scientific questions in astronomy and physics.

### Observations with coherent detection

**Fig. 2.**
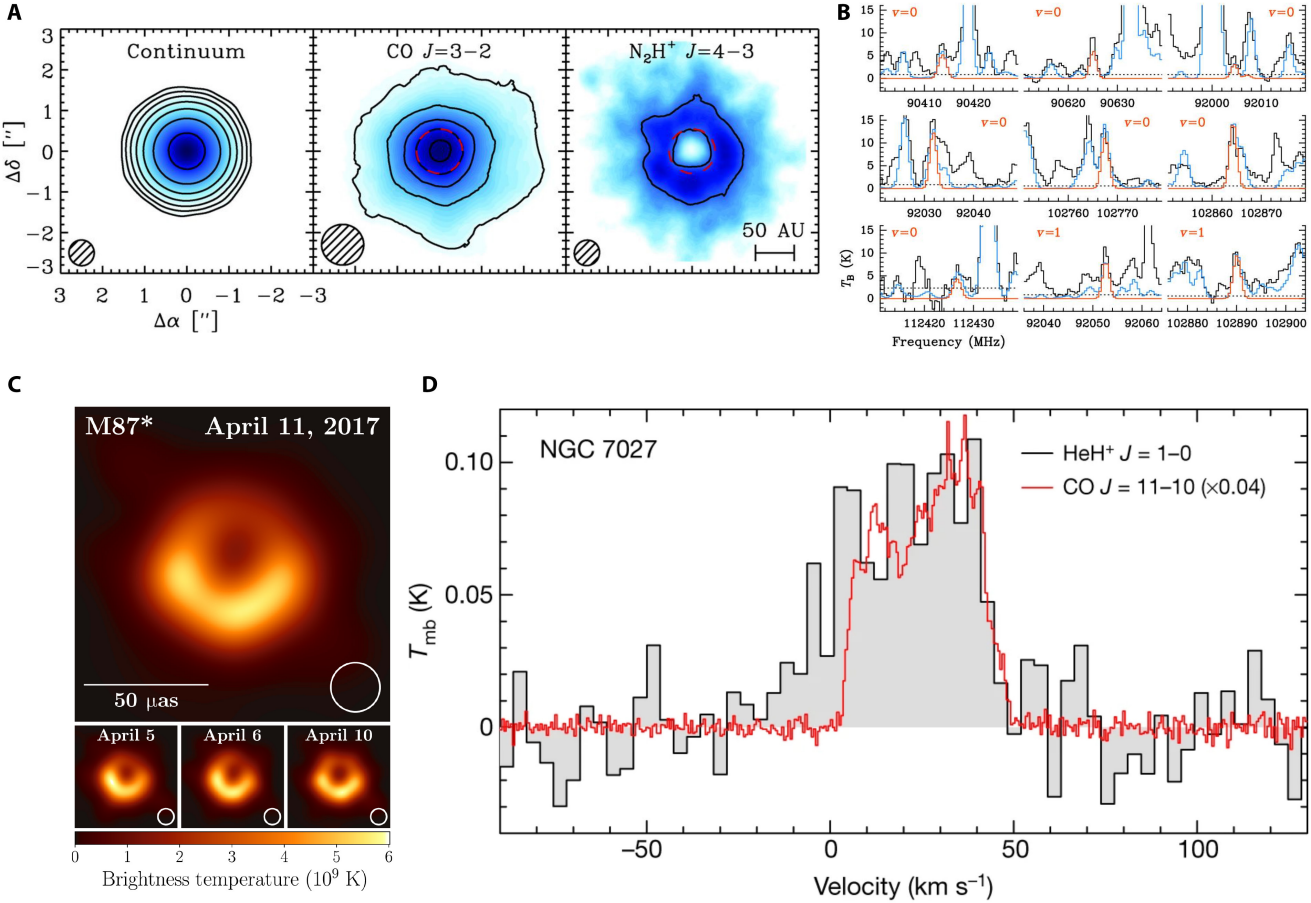
(A) Observed dust, CO, and N_2_H^+^ emission targeting TW Hya [37]. (B) Transitions of NH2C(O)NH2 in either its vibrational ground state or first excited vibrational state observed in the ReMoCA survey toward Sgr B2(N1S) [38]. (C) First image of a black hole from EHT [6]. (D) The HeH^+^ >>J = 1-0 rotational transition spectrum, captured using upGREAT aboard SOFIA, targeting NGC 7027 [7].

The emission lines in the THz band have significantly advanced our understanding of the universe. By probing cold, dense regions of space—often hidden from view in optical wavelengths—using high-resolution molecular lines such as CO (multiples of 115 GHz) [35] and water (557 GHz, 1,661 GHz, and so on) [36], THz observations provide valuable information about the formation and evolution of stars, the complex chemistry of the ISM, the detailed kinematics of the gas, and the origin of life. For example, Qi et al. [37] observed the CO snow line of Neptune with ALMA in 2013, shown in Fig. 2A, helping to identify locations where planet formation occurs. Additionally, Jørgensen et al. [38] detected glycolaldehyde (HCOCH_2_OH), a building block of RNA, at 220 and 690 GHz, suggesting that processes leading to biologically relevant molecules are occurring in space. In 2019, Belloche et al. [39] reported a secure observation of urea, ${\mathrm{NH}}_{2}C\left(O\right){\mathrm{NH}}_{2}$, the start of modern organic chemistry, in a high mass star formation region Sgr B2 (N) as shown in Fig. 2B. In the same year, the Event Horizon Telescope (EHT) revealed the first image of a massive black hole at the center of M87 [6], as shown in Fig. 2C, which was made possible by using very long baseline interferometry (VLBI), which forms a virtual Earth-sized telescope. Furthermore, Güsten et al. [7] observed the helium hydride ion (HeH^+^, 2.011 THz) directly with SOFIA, which is a critical milestone in understanding early cosmic chemistry and the evolution of the universe, as shown in Fig. 2D.

**Fig. 3.**
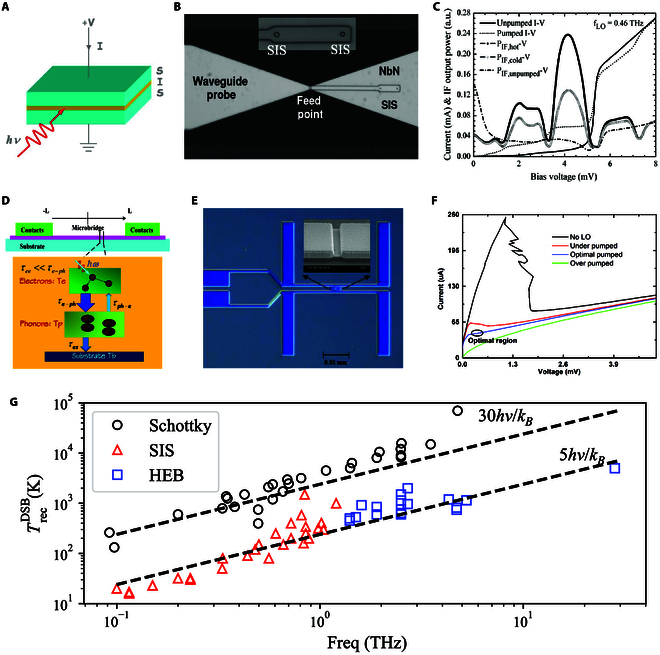
(A) Cartoon representation of an SIS junction. (B) Photo of an NbN SIS mixer [42] with the inset of the SIS junction. (C) Current–voltage (IV) curve of an NbN SIS mixer [42]. (D) Cartoon representation of the hot spot model of the HEB mixer. (E) HEB photo of an antenna-coupled NbN HEB mixer with the inset of the NbN microbridge [51]. (F) IV curve of an NbN HEB mixer [51]. (G) Statistics of the noise temperature of state-of-the-art mixers [48].

These applications require low-noise coherent detectors to measure the amplitude and phase of incoming signals simultaneously. Due to high-performance low-noise amplifiers (LNAs) only available at the lower end of the THz band [40], superconducting heterodyne mixing is currently the key technology enabling such observations. Currently, 2 superconducting mixers are used: the SIS and HEB mixers. Since mixers measure amplitude and phase at the same time, they are subject to the quantum noise limit of $\mathrm{hv}/k_{B}$, originating from the Heisenberg uncertainty principle, where h is the Planck constant, v is the frequency, and $k_{B}$ is the Boltzmann constant. The state-of-the-art performance of these THz coherent mixers is summarized in Fig. 3G.

SIS mixers have long been the dominant technology for high-resolution observations at frequencies below 1 THz. It is a quantum mixer that operates based on the quasiparticle tunneling effect rather than Cooper pair tunneling [41]. An SIS junction consists of 2 superconducting layers separated by an extremely thin insulating barrier (on the order of several nanometers), shown in Fig. 3A and B, which allows quasiparticles to tunnel between the superconductors with the signal. The current–voltage characteristics of the mixer, shown in Fig. 3C, are shaped by this quasiparticle tunneling at V=2Δ/e, below which the current is close to zero. Such strong nonlinearity is the basis that makes SIS mixers highly sensitive for precise millimeter and submillimeter wave detection.

SIS mixers offer several key advantages that make them indispensable in high-frequency astronomy. These include a stable conversion gain, wide instantaneous bandwidth (around 10 GHz), and a relatively low local oscillator (LO) power requirement, typically a few nanowatts. One of the fundamental constraints for SIS mixers is that the working frequency must remain below the energy gap Δ of the superconductor material. If the mixer absorbs photons with an energy large enough (hv>2Δ) to break Cooper pairs, the superconductor becomes lossy, severely degrading the performance.

The operating temperature of SIS mixers is typically set at about half of the superconductor material’s critical temperature ($T_{c}$/2). Nb is the commonly chosen material for SIS mixers for its low radio frequency (RF) loss and mature fabrication, which also makes the SIS mixer operational around 4 K, compatible with cooling technology with 4He. Superconducting materials with higher $T_{c}$ are also preferred to improve the working temperature of the mixer. In 2008, Li et al. [42] from Purple Mountain Observatory (PMO) demonstrated an SIS mixer made of NbN that has a noise temperature close to 5$\mathrm{hv}/k_{B}$ at 460 GHz at temperatures as low as 10 K, which not only extends the working frequency to around 1.6 THz but also significantly reduces the cooling requirements, making SIS mixers suitable for use in extreme environments such as space missions and observatories in Antarctica.

The noise performance of the state-of-the-art SIS mixer made of Nb is close to 2$\mathrm{hv}/k_{B}$ [43], as shown in Fig. 3G. Efforts are continuing to be made to further reduce the noise temperature to improve sensitivity. Future development of SIS mixers is largely focused on scaling the technology to large arrays to improve observation efficiency. Currently, SIS mixer arrays are limited in size, with the largest arrays consisting of 8 × 8 elements. This limitation arises from the complexity of evenly distributing the LO signal across the array and maintaining uniform fabrication quality. There is also an increasing demand for wider intermediate frequency (IF) and RF bandwidths to improve observation efficiency across broader spectral ranges [44]. To this end, efforts are being made to merge multiple observation bands into systems such as ALMA, where more mature SIS fabrication techniques are combined with broadband impedance matching strategies [45].

The dominance of SIS mixers below 200 GHz is currently being challenged by cryogenic LNAs. Cryogenic LNAs based on InP high-electron mobility transistors (HEMTs) up to 115 GHz with a noise temperature of 24 K are mature and commercially available. Varonen et al. [46] reported a cryogenic LNA in the 200 GHz band with a noise temperature of 87 K. Cryogenic LNAs have already been chosen as the first stage for next-generation ALMA band 2 + 3 (65 to 115 GHz) and are being considered for ALMA band 4 + 5 (125 to 211 GHz) [47].

The HEB mixer is the most sensitive mixer above 1 THz, as shown in Fig. 3G. It works as follows [48]. At low temperatures, the electron–phonon interactions in superconductors weaken, as is shown in Fig. 3D, allowing electrons and phonons to be described independently by their effective temperatures. Under the influence of direct current and LO power, usually collected by antennas as shown in Fig. 3E, the electron temperature in the superconducting microbridge rises above the surrounding temperature, forming hot electrons. The material returns to a resistive state as the electron temperature approaches $T_{c}$. This transition creates a sharp change in resistance, shown in Fig. 3F, which can be used to detect even very small changes in the temperature of electrons caused by incoming signals.

HEB is typically composed of NbN, which has an electron–phonon interaction time of approximately 20 ps and a phonon escape time $\tau_{\mathrm{es}}$ (shown in Fig. 3D) of around 50 ps (on a silicon substrate), resulting in an IF bandwidth exceeding 3 to 4 GHz, enough for most applications. With a better understanding of their operating principles [49,50] and improvements in fabrication processes, these mixers now achieve receiver noise temperatures close to 10$\mathrm{hv}/k_{B}$, as shown in Fig. 3G, with some frequencies reaching 5$\mathrm{hv}/k_{B}$ [51]. Ren et al. [52] showed an HEB mixer working at a frequency as high as 28.2 THz with a noise temperature of 3.7$\mathrm{hv}/k_{B}$, indicating that the HEB mixer, in principle, has no upper limit on its operation frequency. For HEB mixers above 2 THz, quantum cascade lasers (QCLs) usually generate the LO signal. Many efforts are being made to integrate the QCL with the HEB mixer. In 2015, Miao et al. [53] demonstrated the integration of a QCL with an HEB mixer, an important step to minimize the system.

One of the ongoing challenges in HEB technology is to increase the IF bandwidth. Currently, the IF bandwidth of HEB mixers made of NbN is significantly narrower than other mixing technologies such as SIS. Expanding the IF bandwidth is essential to improve observation efficiency. Krause et al. [54] expanded the IF bandwidth of the HEB to around 7 GHz by adding a GaN beneath the NbN layer, which improves the phonon matching between NbN and the substrate, leading to faster cooling of the HEB film.

Another ongoing challenge in HEB technology is to scale HEB mixers from single-pixel detectors to large, multi-pixel arrays. The main difficulty lies in the injection of the LO signal. Phase grating is one of the most promising technologies for pumping large HEB mixer arrays [55]. It introduces a spatially periodic phase shift in the transmitted or reflected LO signal and controls the diffraction of THz waves into a specific order.

Materials with higher critical temperatures, such as magnesium diboride (MgB_2_) [49], are being explored for their potential to operate at these elevated temperatures. However, achieving the same level of performance as current NbN HEBs remains a significant challenge.

### Observations with incoherent detection

Many important observations in the THz band require only the magnitude of the signal, such as the detection of CMB, the submillimeter galaxies (SMGs) [56], and new emerging line intensity mapping (LIM) [57].

The CMB radiation, a fundamental pillar of the big bang theory, peaks in the THz frequency range.

It provides a glimpse into the universe around 380,000 years after the big bang, during the era of recombination, when temperatures had dropped enough for protons and electrons to merge, creating neutral hydrogen atoms. This radiation exhibits an almost perfect blackbody spectrum with a temperature of 2.72548 ± 0.00057 K [58]. The CMB exhibits tiny fluctuations, or anisotropies, in $10^{-5}$. These anisotropies were first confirmed by the COBE satellite and later refined by missions such as WMAP and Planck. These tiny fluctuations in the early universe’s radiation reveal the initial density variations that led to galaxy and large-scale structure formation.

By analyzing patterns in CMB, scientists can determine essential cosmological parameters, evaluate the standard model of cosmology, and gain a valuable understanding of dark matter, dark energy, and the curvature of the universe [59]. Additionally, CMB anisotropies offer clues about inflation, neutrino physics, and gravitational lensing effects, making them crucial for verifying general relativity theories and exploring alternative cosmic evolution models.

**Fig. 4.**
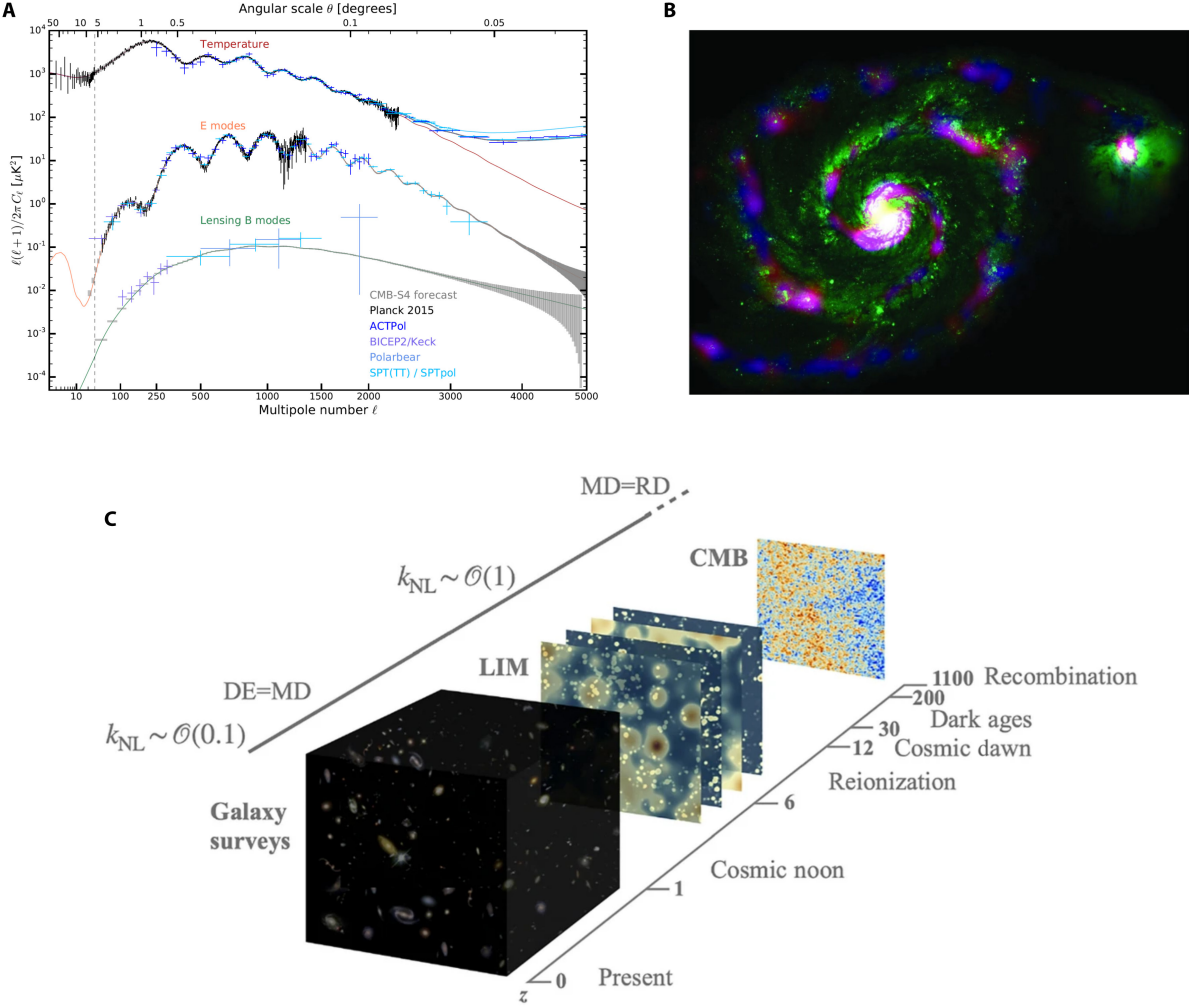
(A) Predicted B-mode from CMB- S4 [59]. (B) Composite image of the iconic Whirlpool Galaxy, combining SCUBA-2 colors—blue representing 450 μm and red representing 850 μm—overlaid on a green-scale Hubble Space Telescope (HST) image [187]. (C) Mapping the intensity of various emission lines opens a window into redshift volumes, offering detailed insights into significant epochs in cosmic history [57].

In recent years, CMB research has increasingly focused on detecting primordial gravitational waves [59], which would provide direct evidence of the rapid inflationary expansion of the universe occurring in the first fractions of a second after the big bang. These gravitational waves would imprint a distinctive “B-mode” pattern in the polarization of the CMB. However, detecting this B-mode signal is extremely challenging as it is buried beneath foreground sources such as emission from interstellar dust and distortions caused by gravitational lensing. Overcoming these obstacles is a key focus of current and future CMB experiments, as shown in Fig. 4A, which requires around 500,000 sensitive detectors.

Another important incoherent observation in astrophysics is the observation of SMGs [56]. SMGs are distant red-shift galaxies that are bright in the THz band, vital for understanding the early universe’s evolution. As some of the most luminous, dust-obscured galaxies, they trace intense star formation, often occurring just 1 to 3 billion years after the big bang, during the peak epoch of star formation. SMGs provide insights into the rapid buildup of stellar mass, galaxy mergers, and chemical enrichment processes, often evolving into massive elliptical galaxies seen today. By penetrating dust clouds, submillimeter observations reveal hidden star-forming regions, contributing significantly to the infrared background and luminosity function, as shown in Fig. 4B.

The last example of incoherent detection is the newly emerging LIM [57] as illustrated in Fig. 4C. Emission lines, such as the CII (1.9 THz)—the brightest emission line from distant galaxies, spread over a broad bandwidth because of the redshift caused by the universe’s expansion. This effect helps trace the evolution of star formation throughout cosmic history. By studying these redshifted lines in the THz band, astronomers can map the star formation rate across different epochs, shedding light on how galaxies evolved over billions of years. The detectors used for incoherent observation are direct detectors that measure only the intensity of the incoming signal. Thus, there is no fundamental limit to their sensitivity. Array detectors are preferred to enhance mapping speed and efficiency, significantly reducing observation time. The 2 leading superconducting detectors in THz astronomy are KIDs [60] and TESs [61], which show background-limited sensitivity and scalability in large arrays.

The main sources of noise from TES are thermal noise, thermal fluctuation noise, photon noise, and readout noise. The thermal noise, or Jonhson–Nyquist noise, is suppressed in a voltage-biased TES. The thermal fluctuation noise, or phonon noise, is proportional to $T^{2}$, where T is the operation temperature of TES since TES works around $T_{c}$ of the used superconductor; thus, by lowering the $T_{c}$ of the used superconductor, the thermal fluctuation noise can be significantly reduced. The materials commonly used for TES are superconductors with a $T_{c}$ range from 50 to 400 mK to minimize the noise, such as tungsten [62] and titanium [63]. A variety of materials are also used for TES by proximity effect, which is the fact that when a normal conductor is placed adjacent to a superconductor, it will also become a superconductor, but with a lower $T_{c}$. Thus, $T_{c}$ of the multilayer superconductor can be tuned by the ratio of the thickness of different layers; this material includes molybdenum-gold (MoAu) [64] and molybdenum-copper (MoCu) [65].

**Fig. 5.**
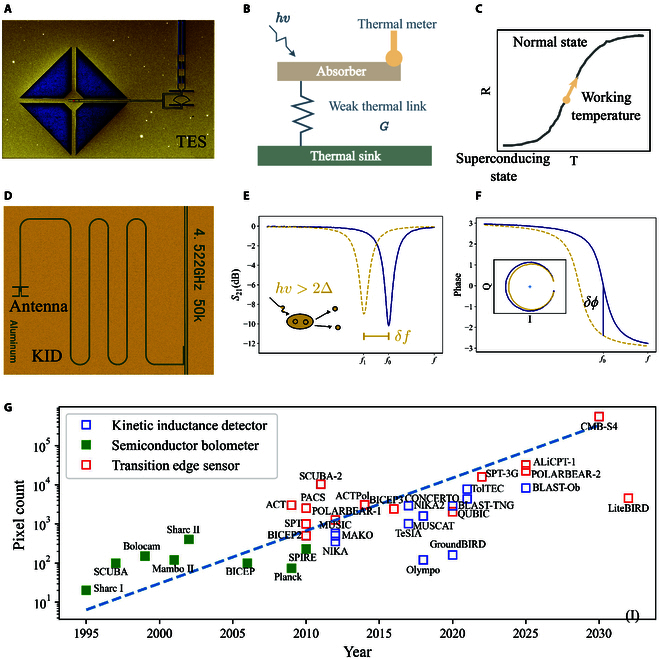
(A) Photo of a typical TES. (B) Cartoon representation of the working principle of TES. (C) Resistance versus temperature of a TES. (D) Photo of an antenna-coupled quarter wavelength KID [188]. (E) Frequency domain response of KID, with the inset showing the Cooper pair breaking by an incident photon. (F) Phase response of a KID, with the inset of the resonator in the inphase-quatrature (IQ) plane. (G) Statistics of pixel count for various projects that used KIDs and TES technologies.

A TES in the THz range is made up of a thin film of superconducting material suspended by thin legs to reduce the thermal coupling to the substrate, as shown in Fig. 5A and B. It is designed to operate within a narrow temperature band just above the threshold between its superconducting and normal states. In this transition region, shown in Fig. 5C, the resistance of the thin film is highly sensitive to minute temperature changes, allowing it to detect even faint photon signals. When biased at a constant voltage, the absorption of photons causes a change in current, which is read using a low-noise superconducting quantum interference device (SQUID) [66], which enables background-limited detection.

One of the main difficulties in fabricating TES is releasing the TESs from the substrate and suspending them by thin legs. In recent years, substantial advancements have been made in fabricating TES detector arrays and developing multiplexed SQUID readout technologies. With improvements in creating highly uniform superconducting thin films in large areas, the technical challenges to producing TES arrays containing thousands of pixels have been largely overcome, as illustrated in Fig. 5G.

Regarding multiplexed readout technology, the most widely used techniques are time division multiplexing (TDM) [67] and frequency division multiplexing (FDM) [68]. Both techniques allow a single readout channel to handle signals from dozens of TES pixels, supporting arrays with hundreds to thousands of pixels. However, both methods have limitations. The readout noise of TDM increases with the square root of the multiplexing ratio, while FDM suffers from high-power consumption and requires complex electronics. These challenges pose difficulties when scaling TDM and FDM for larger arrays with higher multiplexing ratios. Despite these issues, TDM currently provides the lowest readout noise and relatively low power consumption. It is the preferred choice for reading the TES array in space, such as the Athena/X-IFU project [69], which is scheduled to launch in the mid-2030s.

To meet the needs of next-generation TES arrays, microwave multiplexing technology [70] is mature and has emerged as a promising alternative [71]. Microwave multiplexing allows hundreds of TES pixels to be read using one coaxial cable. Optimization efforts are expected to push the multiplexing ratio even higher, potentially reaching the thousand-pixel level. Microwave multiplexing has been selected as the readout technology for CMB-S4, which will have detectors of more than 500,000.

A kinetic induction detector (KID) is a high-quality superconducting resonator [60], either a quarter-wavelength transmission line resonator, as shown in Fig. 5D, or a lumped element composed of a meandered inductor and an interdigital capacitor. When the superconductor absorbs a photon (hv>2Δ), it breaks Cooper pairs into quasiparticles. This process increases the kinetic inductance of the material, reducing the resonance frequency and the quality factor of the resonator, as is shown in Fig. 5E and F. This frequency shift can be detected by sending a probing tone at or near the resonance frequency, allowing the detection of the absorbed photon. One of the primary advantages of KID technology is its natural compatibility with frequency domain multiplexing (FDM).

In a KID array, each detector is tuned to a slightly different resonance frequency, usually separated by 2 to 4 MHz. This frequency spacing enables the simultaneous readout of thousands of detectors over a single coaxial cable.

A comb of signals, each matched to the resonance frequency of a specific detector, enables efficient readout of the entire array, making KID technology exceptionally scalable. This scalability supports large arrays with thousands of pixels, simplifying readout complexity while maintaining performance.

With continued advances in understanding KID physics, their sensitivity has grown markedly over the past 2 decades. A key limitation of KIDs, particularly their low-frequency noise, is due to the 2-level system (TLS) noise [72] at the interface between the superconducting layer and the substrate. Bruno et al. [73] showed that by careful treatment of the substrate surface, the TLS noise can be reduced. Hu et al. [74] and Boussaha et al. [75] showed that operating the KID at a higher temperature can also reduce the impact of TLS. Multiple research groups have shown that KIDs can achieve background-limited sensitivity for ground-based observations since 2011 [76–78]. In 2022, by suspending the aluminum absorber on a membrane, a record sensitivity, Baselmans et al. [79] demonstrated a noise equivalent power (NEP) of $3\times 10^{-20}\;$ W/$\sqrt{\mathrm{Hz}}$ of a KID array at 1.5 THz, which is sufficient for background-limited space observations.

One of the limitations of KIDs is that they can only absorb photons with energies higher than 2Δ. Two primary strategies are being investigated to address this issue. The first involves using materials with a lower Δ, such as multilayer superconductors that take advantage of the proximity effect, in which a normal conductor adjacent to a superconductor acquires superconducting properties. Examples include TiN/Ti/ TiN [80], Al/Ti/ Al [81], and Al/ Au [82]. The second strategy combines bolometer and KID technology to create thermal KIDs (TKIDs), where the absorber is placed on a thermally isolated island, and the temperature change is tracked by a KID. Wandui et al. [83] showed that TKIDs can be background noise-limited for ground-based observations and are less affected by cosmic rays.

The scalability of KID arrays has seen significant advances, with array size exceeding 3,000 pixels, as demonstrated on telescopes like NIKA2 and BLAST-TNG. Liu et al. [84] and Shu et al. [85] showed that post-trimming processes can enhance the yield of the array by reducing frequency collisions, which occur when resonators have similar resonance frequencies that cannot be distinguished. As shown in Fig. 3I, the number of pixels in KID arrays has increased rapidly.

The KID readout system is divided into cryogenic and room-temperature components. The cryogenic section is relatively simple, comprising an LNA and a coaxial cable path, where one coaxial cable can read signals from about 500 to 2,000 KIDs. In contrast, the room-temperature section is more complex, as it involves generating and detecting comb signals at resonance frequencies using a field-programmable gate array (FPGA) [86]. Recent developments in radio frequency system-on-chip (RFSoC) technology have significantly improved this process, highlighting the potential to reduce the cost of the readout system [87].

In recent years, KID has also been shown to be a promising technology for LIM, as demonstrated by CONCERTO [88] and DESHIMA [89]. The main instrument of CONCERTO is a Fourier transform spectrometer (FTS) with 2 KID arrays as detectors. The traveling length and speed of the motor in the FTS limits the spectro-resolution, which is on the order of 100. DESHIMA is an on-chip spectrometer that uses a superconducting filter bank, with each spectral channel detected by a dedicated KID. The filter bank’s quality factor limits the spectro-resolution of the on-chip spectrometer. Laguna et al. [90] show that a filter bank made of microstrip with amorphous silicon (a-Si) as the dielectric can have a quality factor of around 900, promising to improve the spectro-resolution of the on-chip filter, making them a powerful tool for future astronomical and other scientific applications in the THz band.

### Perspective

In the THz band, numerous advanced scientific objectives hold great promise, including obtaining higher-resolution images or even dynamic footage of black holes; exploring the first light of the universe by discovering massive, high-star-formation galaxies in the early universe and verifying cosmological evolution models; conducting submillimeter surveys; studying galaxy clusters to probe cosmic expansion; precisely determining the evolution of the cosmic star formation rate density and baryon fraction; addressing key questions in galaxy evolution, such as the formation and demise of high-redshift extreme galaxies; investigating the ISM and star formation in the nearby universe; examining the circumgalactic medium and baryon recycling in galaxies; exploring molecular clouds and interstellar magnetic fields; studying protoplanetary disks, debris disks, exoplanets, and the origins of life; and advancing submillimeter time-domain astronomy.

The superconducting detectors in the THz band are compared in Table 1, which will continue to be cutting-edge technologies to explore faint signals from the universe as follows.

**Table 1.** Comparison of SIS, HEB, TES, and KID detectors

	SIS	HEB	TES	KID
Type	Coherent	Coherent	Direct detector	Direct detector
Multiplexity	Difficult	Difficult	Easy	Easy
Frequency range	<1THz	>1THz	No limitation	>2Δ
LO power	∼μW	∼nW	/	/
IF bandwidth	∼10GHz	∼4GHz	/	/
Noise temperature	∼4hv/ℏ	∼10hv/ℏ	/	/
NEP	/	/	Background limited	Background limited
Readout	Simple	Simple	Complex	Simple

#### SIS mixer

The future development of the SIS mixer will prioritize expanding both RF and IF bandwidths to enable the detection of a wider range of signals. By broadening the RF and IF bandwidths, SIS mixers will enhance their capability to process broader spectral information, which is crucial for capturing intricate astronomical signals and improving observational flexibility. In addition, increasing the size of the array will increase the sensitivity and spatial resolution, making SIS mixers more effective. Efforts will also focus on reducing the noise to approach the quantum noise limit, enhancing the mixer’s sensitivity to faint signals. The InP HEMT cryogenic amplifier will challenge the dominance of the SIS below 200 GHz.

#### Hot electron bolometer

HEB will continue to be the most sensitive detector above 1 THz. The future development of HEB will focus on several key enhancements to boost its performance in astronomical and scientific applications. One priority is to enlarge the array size, enabling more efficient observations. Expanding the IF bandwidth is also a major goal, allowing HEBs to process a broader range of signals, thereby increasing their utility in wideband spectroscopy. Efforts will be directed toward reducing the noise of HEB to be closer to the quantum noise limit. Furthermore, advances will aim to operate HEBs at higher temperatures using high-$T_{c}$ materials, reducing the cooling demands and making them more practical for a wider range of applications.

#### Transition edge sensor

The development of microwave multiplexing has significantly simplified the TES readout system, reducing its complexity and cost. As shown in Fig. 3I, the sizes of the TES array have steadily increased, with 100,000-pixel focal plane arrays anticipated by the 2030s. Recent advances in quantum parametric traveling wave amplifiers (QPTAs) also offer the potential for further reductions in readout noise. Although QPTAs are still being developed, their future scalability and improved fabrication techniques could enable microwave multiplexing noise levels comparable to those of time-domain multiplexing.

#### Kinetic inductance detector

KIDs are emerging as a strong competitor to TES in terms of sensitivity and scalability. TES detectors have an advantage because of their multichromic capability, which simplifies front-end optics by eliminating the need for a dichroic filter. However, there are active efforts to develop multichroic KID arrays, particularly for CMB observations [91,92].

One challenge for KIDs is their sensitivity, often limited by the TLS noise caused by the dielectric materials used in microstrip lines, a problem not typically encountered in TES systems. Recent studies suggest that a-Si could be an effective dielectric to minimize TLS noise, potentially enhancing KID performance in multiband observations like CMB experiments. As a result, the KID array sizes are likely to increase further, potentially matching the scale of the TES arrays.

## THz Frequency Source and Communication Systems

### THz Schottky diodes and frequency multiplier

THz frequency sources are indispensable for these application systems and directly affect performance. According to the principle of signal generation, THz sources are divided into 2 categories [8,93,94]: photonics and electronics. Although photonic THz sources have advantages such as good coherence and room-temperature operation, their low energy conversion efficiency and complex structure limit their applications. Electronic THz sources, especially those composed of solid-state semiconductor devices, have become the mainstream research direction due to their easy integration, small size, and low cost. As a core device, Schottky diodes are widely used in THz frequency multipliers due to their high frequency, high integration, and low power consumption, which have promoted the rapid development of THz technology.

#### THz Schottky diodes and frequency multiplier

**Fig. 6.**
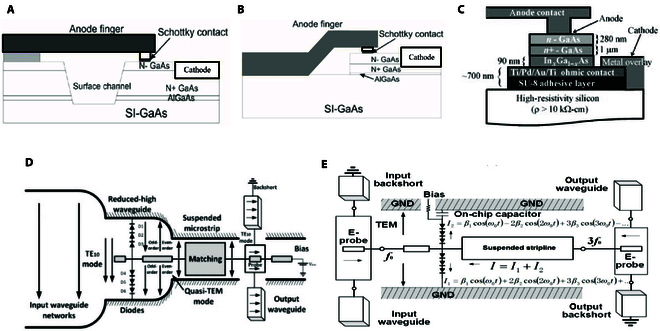
(A) Surface channel-etched diode [96]. (B) Air-bridge planar diode [97]. (C) Quasi-vertical structured Schottky diode [95]. (D) Balanced doubler [98]. (E) Balanced tripler.

The mainstream Schottky diodes can be categorized into 3 types: surface channel-etched planar Schottky diodes, planar diodes with air-bridge structure, and quasi-vertical Schottky diodes, the basic structures of which are displayed in Fig. 6A to C [95–97]. Based on these high-performance Schottky diodes, Schottky multipliers have advanced rapidly in recent years.

The theory of THz balanced doubler was first proposed by Erickson et al. [98] in the United States in 1993, which is illustrated in Fig. 6D. This structure realizes that the intrinsic suppression of odd harmonics through the reverse loading of the input signal in the isotropic shunt diode, which effectively improves the frequency conversion efficiency, the compact configuration of this circuit, and the high degree of isolation of the input–output signals, is conducive to the independence of matching circuits, which makes it the most commonly used balanced 2-octave circuit design structure at present.

The balanced structure of the THz tripler shown in Fig. 6C utilizes reverse parallel Schottky diode pairs to construct an idle loop for even harmonics, which can achieve the intrinsic suppression of even harmonics and ensure the balanced output of odd harmonics, which is superior to the unbalanced structure in terms of bandwidth and frequency doubling efficiency but requires additional on-chip capacitance (usually tens of nanometers of silicon nitride or silica material) to realize the dc power supply and RF grounding. The existence of on-chip capacitance brings 2 difficulties: one is that the on-chip capacitance needs to be realized using a monolithic process, which has low process compatibility and demanding assembly requirements; the other is that on-chip capacitance brings additional insertion loss, which makes the high harmonics not ideal grounded in this capacitor [98]; thus, the overall amplitude and phase of the circuit are unbalanced, which ultimately leads to decrease in efficiency. However, along with the maturity of the monolithic process, the balanced triplex circuit structure has been widely studied and applied in recent years.

#### GaAs-based and GaN-based Schottky diode frequency multiplier

Gallium arsenide (GaAs) is a widely employed semiconductor material, known for its high electron mobility, low noise characteristics, and strong resistance to radiation. In addition, its manufacturing process is well developed. These attributes make GaAs one of the most popular materials in the semiconductor industry. In the THz domain, Schottky diodes play a critical role, particularly in frequency multiplier designs, due to their efficiency in generating higher-frequency signals. With advancements in material science and semiconductor technology, terahertz monolithic integrated circuits (TMICs) have emerged as promising solutions for frequency multiplication in THz applications. TMICs leverage the properties of GaAs to offer enhanced performance in high-frequency operations.

**Fig. 7.**
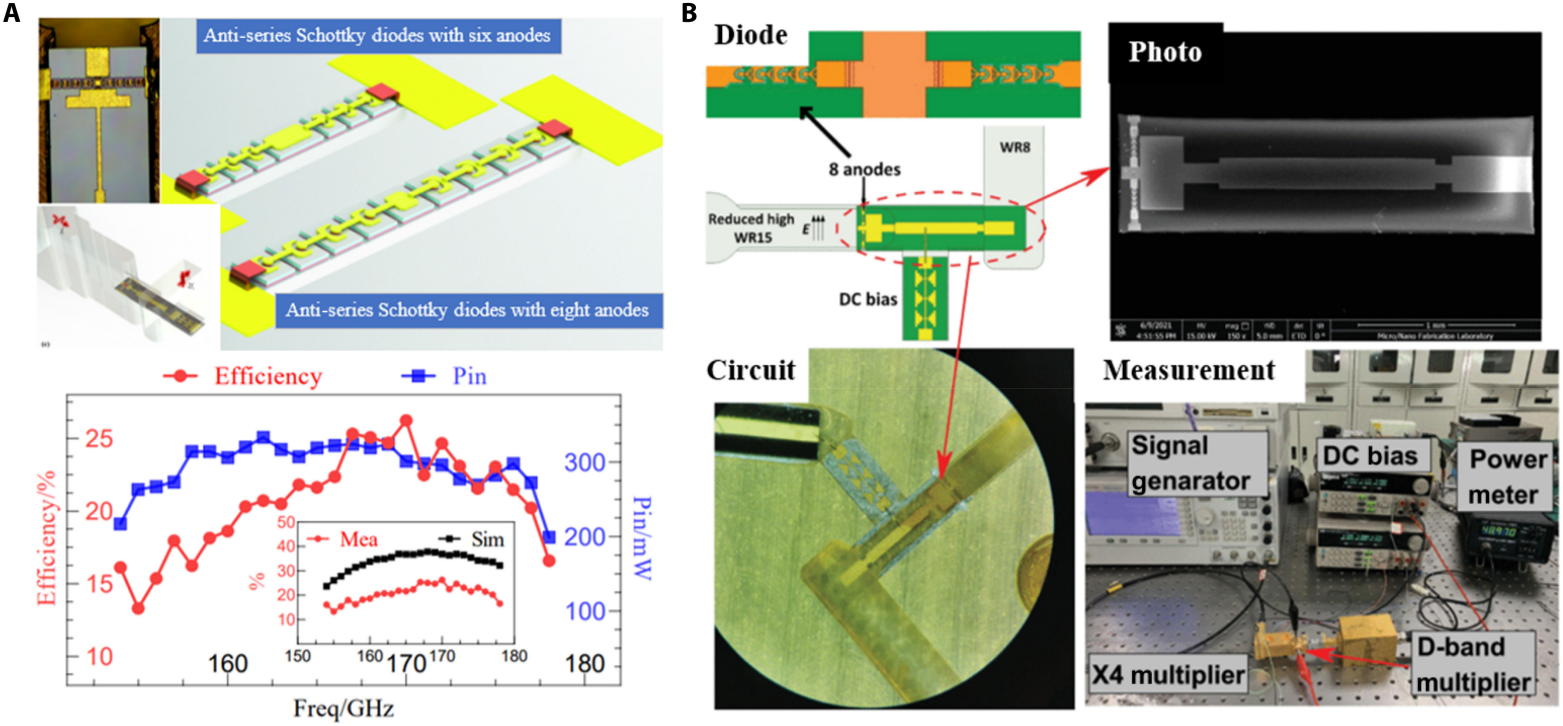
(A) The 170- and 340-GHz diode multiplier [100]. (B) D-band GaN-based diode multiplier [103].

In 2016, ACST (Germany) reported developing a series of balanced monolithic integrated frequency multipliers using a thin-film diode process. These devices included diodes and multipliers operating at various frequencies: a 332 GHz diode, a 440 GHz diode, a 332 GHz tripler, and a 660 GHz tripler, achieving peak output powers of 14, 12, 8, and 5 mW, respectively. The thin-film process used in these devices is a key approach in designing GaAs-based monolithic integrated multipliers [99]. Research on GaAs monolithic integrated frequency doubler circuits has also advanced significantly in China. In 2021, Zhou et al. [100] fabricated 2 monolithic integrated diodes on 15-μm-thick GaAs thin-film substrates. These diodes operated at 170 and 340 GHz, with configurations of 8 anodes for the 170 GHz diode and 6 anodes for the 340 GHz diode. As shown in Fig. 7A, these multipliers demonstrated peak efficiencies of 26.25% at 170 GHz and 46% at 341 GHz. More recently, in 2023, Song et al. [101] from China Electronics Technology Group (CETG) developed a high-performance GaAs-based frequency doubler, which achieved output powers of 42 to 78 mW in the 170 to 220 GHz range, with efficiencies between 14% and 26%.

In addition to its advantages, GaAs has some limitations, such as a lower breakdown voltage and poor thermal conductivity. These factors restrict its use in high-power THz devices, especially in THz frequency doublers with multi-anode structures, where managing heat becomes a significant challenge [102]. In contrast, GaN offers several advantages over GaAs. It has a wider electronic bandgap, higher thermal conductivity, and higher breakdown voltage, making it more suitable for handling higher power and improving thermal management [23]. Because of these characteristics, GaN-based Schottky diodes have gained popularity in high-power frequency doubler circuit designs in recent years, showing strong potential for further development.

In 2022, Gioia et al. [24] reported the development of a GaN Schottky diode fabricated with low series resistance, making it suitable for the design of THz frequency doubling circuits. There have also been significant domestic reports on GaN high-power frequency doubling circuits. In 2022, Zeng and colleagues [103] of the China Academy of Engineering Physics (CAEP) introduced a high-efficiency integrated GaN monolithic D band diplexer with strong power handling capabilities shown in Fig. 7B. The GaN Schottky structure was formed on a sapphire substrate by chemical vapor deposition. The frequency multiplier demonstrated the ability to withstand up to 0.5 W of continuous-wave input power, achieving a maximum conversion efficiency of 17% at 115.6 GHz and an output power of 286 mW. In 2023, Luo and Zhang [25] exhibited a monolithic integrated broadband G-band diplexer based on reverse series planar GaN Schottky diodes. These GaN diodes were fabricated on SiC substrates to enhance heat dissipation. The simulation results indicated that the diplexer could produce maximum output powers of 97 and 150 mW when the input powers were 500 and 750 mW, respectively. These studies indicate significant progress in domestic research on GaN high-power frequency doublers, with output power reaching the watt level.

#### Perspective

**Fig. 8.**
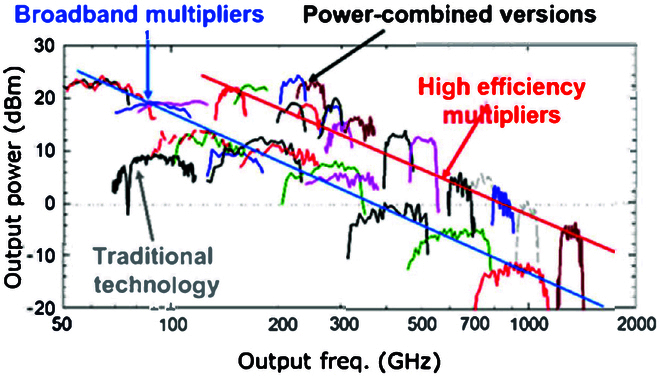
Comparative performance of high-power and broadband sources within 2 THz frequency range [8].

The planar Schottky diode is currently the most widely used core nonlinear device in THz frequency sources, owing to its ease of integration, stability, and reliability. GaAs, with its unmatched high electron mobility, remains the primary substrate material for THz high-frequency Schottky diodes. However, gallium nitride is gaining attention for its superior power handling capabilities in high-power frequency doubling circuits. In this field, both discrete and monolithic integrated circuits coexist, but as researchers delve deeper into the THz high-frequency spectrum, monolithic balanced integrated circuits are expected to offer more advantages. Figure 8 summarizes the comparative performance of the Schottky diode-based high-power frequency source versus the broadband frequency source within the 2 THz spectrum [8]. The comparison reveals a notable parallel disparity: While the operational frequency range of the broadband frequency source encompasses roughly half of the entire waveguide rectangular (WR) band, its output power is approximately 10 dB lower than that of the high-power frequency source. This highlights a trade-off between the Schottky diode-based frequency multiplier’s operational bandwidth and output power. In response to this, new design methods and circuit configurations are emerging for high-power multipliers with broad operational frequency ranges, driving advances in THz frequency sources. These improvements will improve output power, operating bandwidth, frequency-doubling efficiency, stability, reliability, and miniaturization while reducing cost and power consumption. As semiconductor technology progresses, novel materials—such as new III–V compounds and heterogeneous integrated structures—will further optimize Schottky diode production and frequency-doubling circuits. This will enhance the performance of THz frequency sources, leading to more powerful superheterodyne receivers that expand applications in detection, metrology, imaging, and communication. In turn, these advances will accelerate the overall development of THz technology, making it more widely accessible and impactful across various fields.

### THz communication systems

#### Fully electronic systems

**Fig. 9.**
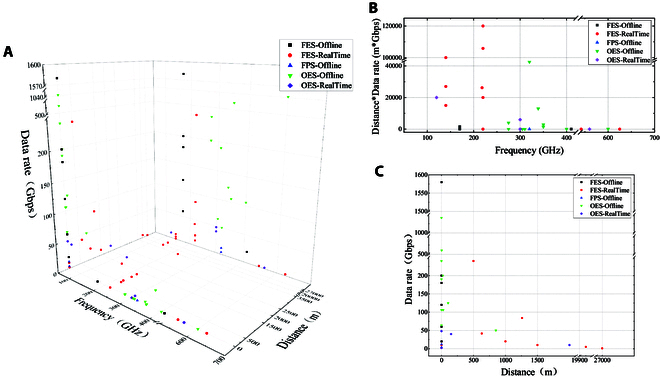
Schematic diagram of the communication prototype with frequency, distance, and rate as coordinates, respectively: (A) 3-dimensional figure; (B) distance rate product–frequency; and (C) data rate–distance.

Based on the advance of the THz frequency multiplier, mixer, and other devices, significant progress has been made in THz communication systems, particularly in device development, power source design, channel modeling, and high-speed data transmission. Some prototypes have even been successfully implemented in real-world applications. A summary of representative communication prototypes is provided in Table 2 and visualized in Fig. 9, with the corresponding visual illustrations presented in [19]. THz communication systems are primarily built on 3 key technologies: fully electronic systems (FESs), optoelectronic hybrid systems (OESs), and fully photonic systems (FPSs). Recent advancements in THz communication have driven the field toward achieving higher capacity, wider bandwidth, longer transmission distances, reduced power consumption, and improved integration. Fully electronic THz communication systems offer advantages such as compact structure, high integration, and low power consumption while supporting high-order quadrature amplitude modulation (QAM) schemes. The core approach involves generating a modulated IF signal at the baseband, which is then upconverted to the THz band using a solid-state THz mixer. The signal is effectively transmitted through a solid-state THz amplifier and a high-gain antenna. On the receiving end, the THz signal is down-converted to IF, followed by demodulation and further signal processing.

**Table 2.** Summary of recent THz telecommunication developments

Ref	Year	System indicators							
		System	Scenario	Frequency	Data rate	Distance	Modulation	Multiplex	Methods
[19]	2008	OES	Outdoor	120 GHz	10 Gbps	2 km	/	/	Real-time
[20]	2011	FES	Indoor	625 GHz	2.5 Gbps	cm-level	/	/	Real-time
[21]	2011	FES	Outdoor	140 GHz	10 Gbps	1.5 km	16QAM	/	Offline/real-time
[22]	2012	FES	Indoor	542 GHz	2/3 Gbps	1 cm	ASK	/	Real-time
[104]	2017	FES	Outdoor	140 GHz	5 Gbps	20 km	16QAM	/	Real-time
[105]	2020	FES	Marine	140 GHz	1 Gbps	27 km	QPSK	/	Real-time
[106]	2021	FES	Air-Ground	220 GHz	20 Gbps	1 km	16QAM	/	Real-time
[190]	2021	OES	Indoor	300 GHz	2×24 Gbps	3 cm	OOK	FDM	Real-time
[107]	2022	FES	Outdoor	220 GHz	240 Gbps	500 m	16/32/64QAM	PDM + SDM	Real-time
			Indoor		/	3.5 km	QPSK		
					10 Gbps	0.6 m	64QAM	SDM	Real-time
[110]	2022	OES	Outdoor	300 GHz	2×20 Gbps	150 m	16QAM	FDD/TDD	Real-time
					1 Gbps				
[115]	2022	OES	Indoor	350 GHz	106 Gbps	26.8 m	16QAM	/	Offline
					510.5 Gbps	2.8 m	64QAM	FDM + PDM	
				400 GHz	106 Gbps	0.5 m	16QAM	/	
[111]	2022	OES	Outdoor	339 GHz	124.8 Gbps	104 m	256QAM	/	Offline
[189]	2023	OES	Outdoor	320 GHz	50 Gbps	850 m	16QAM	/	Offline
[114]	2023	OES	Indoor	560 GHz	2 Gbps	0.6 m	OOK	/	Real-time
[113]	2023	OES	Indoor	600 GHz	1.041 Tbps	cm-level	16/32/64QAM	FDM	Offline
[108]	2023	FES	Indoor	110–170 GHz	60 Gbps	15 cm	BPSK	/	Offline
					120 Gbps		QPSK		
					180 Gbps		16QAM		
					200 Gbps		32QAM		
[109]	2023	FES	Indoor	410 GHz	20 Gbps	1 m	QPSK	/	Offline
[190]	2023	FES	Outdoor	198 GHz	2×20.8 Gbps	630 m	16QAM	FDM	Real-time
				218 GHz					
[191]	2024	FES	Outdoor	220 GHz	84 Gbps	1.26 km	16QAM	FDM	Real-time
[116]	2024	OES	On-chip	305 GHz	2×32 Gbps	cm-level	OOK	PDM	Offline
				310 GHz	190 Gbps		16QAM		
[118]	2024	FPS	Indoor	120–320 GHz	12 Gbps	1 m	QPSK	/	Offline
					4 Gbps				
[112]	2024	OES	Indoor	275 GHz	240 Gbps	30 mm	64QAM	/	Offline
					200 Gbps	20 m	32QAM		
[117]	2024	FES	Indoor	135–170 GHz	1.58 Tbps	1 m	16/64QAM	OAM + PDM + FDM	Offline

Research on THz communication technology began internationally in the early 2000s. In 2011, Bell Labs conducted an error-free signal transmission experiment at 2.5 Gbps using a 625 GHz carrier frequency, employing Schottky diode technology at the receiver [20]. The Tokyo Institute of Technology used resonant tunneling diodes (RTDs) for direct intensity modulation and wireless data transmission [22]. They demonstrated direct amplitude shift keying (ASK) wireless data transmission using RTDs, achieving a communication rate of 2 Gbps with a bit error rate of $2\times 10^{-8}$.

**Fig. 10.**
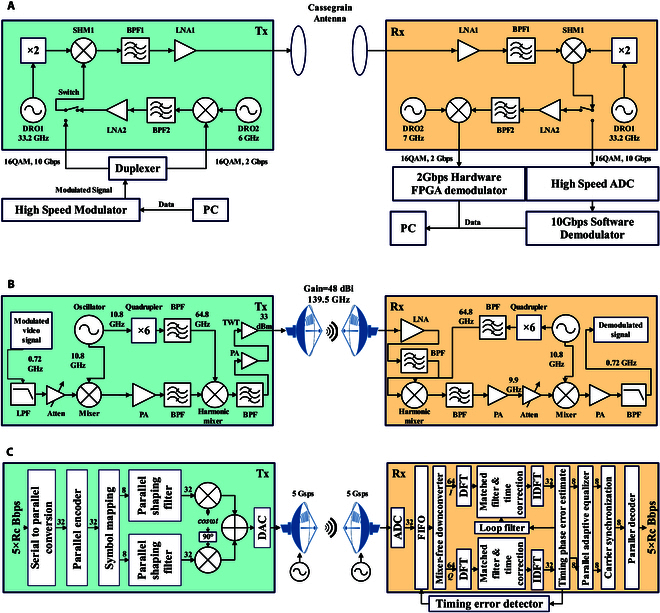
Block diagram of the 140 GHz long-range THz communication system of the Chinese Academy of Engineering Physics: (A) 1.5-km communication system wireless link schematic [21]; (B) 21-km baseband signal processing architecture block diagram [104]; (C) 27-km THz wireless communication system block diagram [105].

The CAEP, one of China’s earliest teams to explore THz technology, has made remarkable achievements in fully electronic THz communication systems. In 2011, they successfully developed a high-speed THz wireless communication system of 0.14 THz, 16QAM, 10 Gbps, marking China’s first kilometer-scale ultra-high-speed THz wireless communication prototype [21]. The block diagram of the transceiver link is shown in Fig. 10A. In 2017 and 2020, they completed high-speed wireless transmission experiments over distances exceeding 20 km in the 140 GHz band [104] and a 27-km high-speed wireless video transmission experiment across a sea surface [105]. The wireless link diagrams are illustrated in Fig. 10B and C. In 2021, they conducted dynamic technology verification for airborne high-speed communication in the 220 GHz band [106]. This was the first verification of a 220 GHz band wireless communication system based on an airborne platform, marking a pivotal transition from ground-based static validation to airborne dynamic validation for THz wireless communication systems.

In 2022, Huawei developed an integrated ultra-broadband THz communication sensing platform operating at 220 GHz, supporting data rates up to 240 Gbps and millimeter-level high-resolution sensing [107]. This demonstrated the feasibility of THz technology for integrated communication and sensing applications in 6G. Addressing issues such as THz RF imperfections, inconsistent front-end performance, and signal crosstalk, in 2023, the University of Electronic Science and Technology of China (UESTC) proposed a 220 GHz fully solid-state THz full-duplex communication system using FDM and orthogonal FDM (OFDM) [108]. This system can operate in complex and unstable environments, such as high humidity or foggy conditions, where channel stability is compromised. Subsequently, breakthroughs were made in overcoming the bottlenecks of system integration, baseband signal processing, and RF damage in THz communication systems. A 220 GHz communication system was developed, which achieved an air interface rate of 84 Gbps over a distance of 1.26 km [109]. This system was successfully applied during the 31st Chengdu Universiade in 2023, enabling real-time transmission of uncompressed 8K ultrahigh-definition video, marking a significant leap from experimental THz systems to practical applications.

#### Optoelectronic hybrid systems

Given that THz waves have a lower frequency than optical waves, generating and controlling THz waves using optical techniques is relatively simpler. As a result, early research on THz communication systems predominantly employed optoelectronic hybrid technology. This approach utilizes the nonlinear properties of optical modulators to generate coherent high-order optical sidebands. These sidebands are then mixed in a photodetector to produce a modulated THz signal transmitted via an antenna. On the receiving end, a THz heterodyne receiver downconverts the THz signal to the IF band, followed by demodulation and signal processing.

Nippon Telegraph and Telephone Corporation (NTT) developed a 120 GHz band, 10 Gbps wireless transmission system using photonic and fully electronic technologies. It was successfully tested in a live trial during the 2008 Beijing Olympics, validating its feasibility in real-world applications [19]. With advances in semiconductor technology, in 2022, the Technical University of Braunschweig, as part of the Horizon 2020 project “ThoR”, established the first bidirectional 300 GHz backhaul end-to-end communication link [110]. Using an RF front-end based on InGaAs mHEMT technology and a spurious-free, low phase-noise photonic solution, this system achieved a net data rate of 2 × 20 Gbps over a 150-m distance. Furthermore, it confirmed the applicability of the IEEE Std 802.15.3-2023 protocol in this context.

**Fig. 11.**
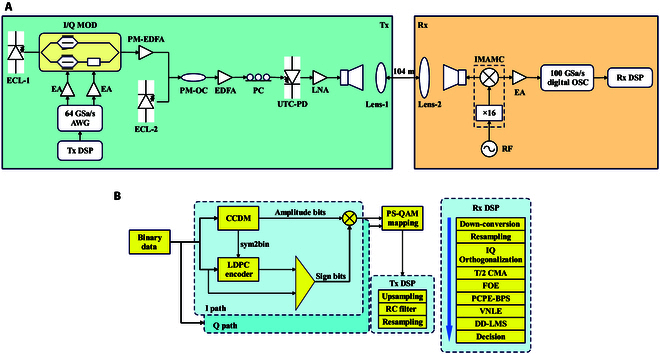
Demonstration of an optoelectronic hybrid THz communication system at Fudan University [111]: (A) an experimental setup for a wireless transmission system of 124.8-Gbps THz signals; (B) a 124.8-Gbps PS-QAM generation scheme and a digital signal processing (DSP) program for the Tx/Rx side.

In 2022, Fudan University experimentally demonstrated a THz wireless communication system assisted by photonics operating at 339 GHz, achieving the transmission of a probabilistically shaped 124.8 Gbps 256QAM signal (PS-256QAM) over a wireless distance of 104 m [111]. This marked the first successful transmission of a single-carrier THz signal over a distance greater than 100 m at a data rate exceeding 100 Gbps, setting a record for net spectral efficiency at 6.2 bit/s/Hz. The system block diagram is shown in Fig. 11A, while Fig. 11B illustrates the corresponding PS-QAM generation scheme and transceiver signal processing workflow. To further showcase the high-speed potential of THz communication, Osaka University introduced an ultra-low phase-noise transmitter and receiver in 2024, employing photonic technology to create a 275 GHz sub-THz wireless link [112]. This link achieved a record-breaking single-channel data rate of 240 Gbps and successfully transmitted 200 Gbps over 20 m.

It is evident that most practical systems currently operate below the 400 GHz band, with only a few studies exploring frequencies above 400 GHz. Since the 300 GHz band offers only about 120 GHz of bandwidth, achieving Tbps systems presents significant challenges. Based on the above analysis and the impact of atmospheric absorption attenuation, the 600-GHz band has garnered increasing attention from researchers due to its large bandwidth and relatively low atmospheric loss. In 2023, the University of Lille in France demonstrated a data rate exceeding 1 Tbit/s using waveguide-integrated THz photodiodes and waveguide receivers in the 600 GHz band, achieving the highest data rate in this frequency range [113]. Similarly, Tokushima University showcased a promising solution for high-frequency THz communication in 6G using a 560 GHz wireless communication system based on a Kerr microresonator frequency comb, achieving 2 Gbps on–off keying (OOK) data transmission [114].

One method of increasing communication capacity in THz communication systems is polarization multiplexing. The Royal Institute of Technology (KTH) in Sweden achieved a total raw data rate of up to 612.65 Gbps in the 350 GHz band by combining frequency and polarization multiplexing [115]. A key component in polarization multiplexing is the polarization multiplexer; however, existing planar multiplexers lack the ultra-wideband capabilities needed for THz frequencies. To address this, in 2024, the University of Adelaide proposed an integrated ultra-wideband THz polarization multiplexer based on a fully silicon-effective medium cladding [116]. Using this multiplexer, they demonstrated real-time dual-channel video transmission at 300 GHz, achieving 80 and 75 Gbps single-channel 16QAM modulation communication at the 310 GHz band. In addition to polarization multiplexing, orbital angular momentum (OAM) multiplexing can enhance channel capacity in THz communication. OAM multiplexing allows for the transmission of multiple independent data streams using OAM beams, and even in line-of-sight (LoS) environments, spatial multiplexing gains can be achieved by increasing the number of available OAM modes. NTT demonstrated the highest publicly reported wireless data rate of 1.58 Tbps in the sub-THz band using OAM multiplexing based on a broadband Butler matrix for 6G backhaul and fronthaul networks [117]. Photonic technology is expected to play an increasingly crucial role in the transition to a full-spectrum communication paradigm.

#### Fully photonic systems

**Fig. 12.**
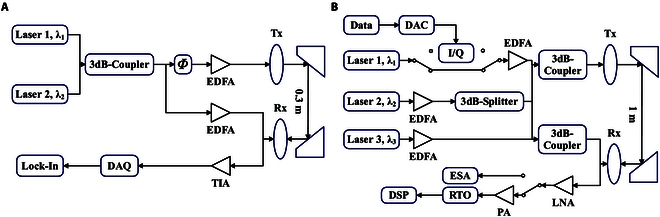
Measurement device developed by HHI for characterizing outlier receivers [118]: (A) a homologous configuration in which 2 lasers generate one optical hop for transmission and reception; (B) an outlier configuration in which 3 lasers generate 2 hops for Tx and Rx, respectively.

To date, most photonic-assisted THz communication links have utilized optoelectronic technology exclusively at the transmitter, leaving the full potential of photonic THz communication untapped. Recognizing this opportunity, the Fraunhofer Heinrich Hertz Institute (HHI) unveiled a heterodyne THz receiver in 2024, employing photonic mixing for communication links operating in the 100 to 300 GHz range [118]. The details of the implementation are shown in Fig. 12. This innovative system successfully demonstrated error-free transmission of 4QAM signals at data rates of up to 12 Gbps within a fully photonic wireless framework. While the current data rate and conversion gain fall short of those achieved by advanced electronic receivers, the latter face inherent limitations due to narrower carrier bandwidths, making them incapable of covering the expansive 100 to 300 GHz spectrum with a single device. Enhancing the link budget in FPSs could further narrow this performance gap. Moreover, photonic THz links seamlessly integrate with existing optical fiber communication infrastructure, underscoring their practical applicability. This pioneering achievement highlights the promise of optoelectronic receivers in the advancement of THz wireless communication and sets the stage for deeper exploration into fully photonic THz links.

#### Perspective

Significant progress has been made in THz communication systems worldwide, with key achievements originating in countries such as the United States, Germany, China, and Japan. An analysis of link performance, technological implementations, and operational frequency bands is as follows.

First, from the perspective of single-link communication metrics, the core objectives of THz communication are to enhance capacity and extend communication distance to meet diverse application needs. Existing systems have achieved transmission rates from tens to hundreds of Gbps, and countries are striving to break the Tbps barrier, with a particular focus on long-distance applications, especially those exceeding kilometer-scale transmissions.

Second, about technological implementation, the current mainstream approach is FESs based on semiconductors. Optoelectronic hybrid technologies are gradually gaining attention because of their compatibility with optical fiber communications and the advantage of low-phase noise in signal generation. FPSs, although promising ultra-high bandwidth potential, face challenges due to the relatively underdeveloped state of photonic receiver technologies. Each of the 3 primary implementation methods, fully electronic, optoelectronic hybrid, and fully photonic, has its strengths and weaknesses, as detailed in Table 3, allowing flexible selection and combination based on different application scenarios.

**Table 3.** Comparison of different approaches of THz communications

Method	FES	OES	FPS
Signal generation	Semiconductor electronic devices	Photomixing/optoelectronic mixing	Photomixing/difference frequency technology
Modulation/demodulation	High-speed mixers/switches	Photodetectors and mixed optoelectronic signal processing	All-optical high-speed modulators
Signal transmission	Antenna	Antenna/optical fiber	Optical fiber/antenna
Advantages	Mature electronic component technology, high technical compatibility	Low phase noise, suitable for high-speed data rates, compatible with existing optical fiber infrastructure	Ultra-high data rates and ultra-wide bandwidth, suitable for integration with existing optical fiber infrastructure
Disadvantages	Performance degrades at higher frequencies, limited power output, large phase noise	Lower output power, complex system, involves optoelectronic conversion, high cost	High system complexity, expensive, sensitive to environmental conditions

The U.S. and Europe concentrate on the 0.2 to 0.4 THz range, while Japan has made notable progress in the 0.3 to 0.72 THz range. However, there have been no public demonstrations of communication systems operating above 1 THz, the so-called “true THz” band. Despite devices supporting these frequencies, challenges such as low power, high noise, complex channels, and severe attenuation have hindered further development. With continued technological advances, communication systems operating in the “true THz” band are expected to become a reality.

Although THz communication has made initial breakthroughs, it still faces challenges before large-scale commercialization. These challenges stem from 2 main factors: First, the foundational scientific understanding of the THz band is still limited, particularly regarding channel models and transmission characteristics. Second, key components, such as high-power, low-noise devices, have yet to overcome significant technical bottlenecks. Continuing efforts in foundational science and key technologies are necessary to propel THz communication toward commercialization.

## THz Biophysics

THz wave-based physical regulation leverages the unique physical properties of THz waves to influence the structures and functions of biological macromolecules and ultimately achieve desired physiological functionalities. It attracts increasing attention due to nonthermal, noninvasive, and reversible modulation manners, showing great potential in biomedicine and brain sciences. However, for THz technology to serve human health, in addition to developing THz sources and intervention means to achieve spatially precise stimulation and improve detection sensitivity, the most important issue we need to address is biosafety. This is because biological systems, particularly nervous systems, are multi-level structures with complex signal transduction networks and positive and negative feedback regulatory loops, making the complexity of neuromodulation unprecedented. As a result, we must fully understand the underlying regulatory mechanisms of THz waves to minimize side effects as much as possible in future practice. The review is organized according to different biological components that are the basis for all physiological activities. Understanding and summarizing the physical laws behind the THz regulation of different biological components not only is conducive to laying a theoretical framework for this emerging discipline and forming a universal law expanded to all aspects of biological systems but also enlightens scientists to develop appropriate THz sources and detection technologies for future applications.

### THz bioeffects on different biological components

**Fig. 13.**
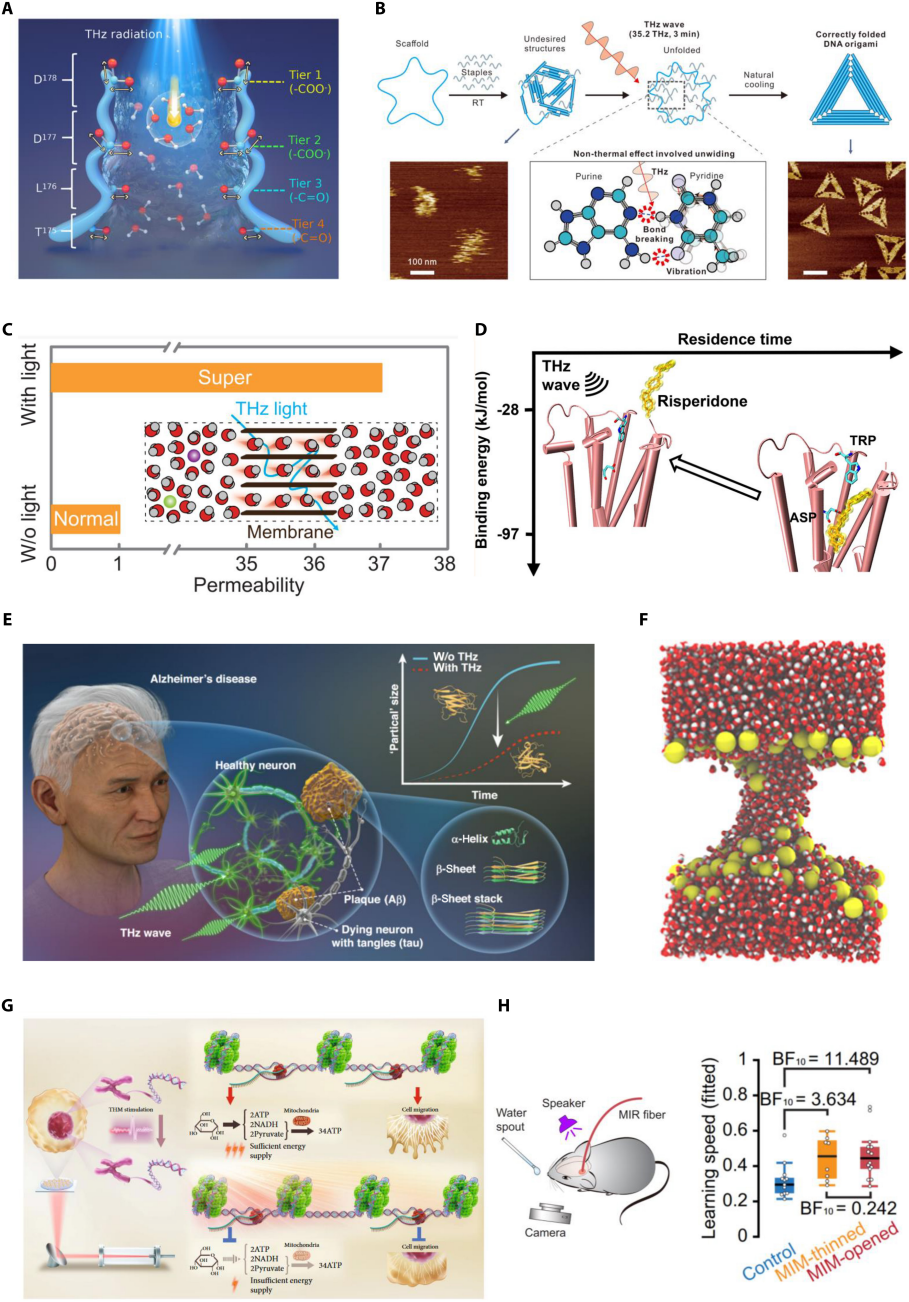
Impact of frequency-specific THz radiation on (A) the permeability of ion channels [119], (B) unwinding of DNA [138], (C) permeability of water channels [147], (D) binding affinity between the receptor and ligand [153], (E) protein secondary and tertiary structures [162], (F) membrane performation [169], (G) cell migration [141], and (H) animal learning speed [121].

A series of theoretical simulations and experimental verifications have been performed at biological or biomimetic components, clarifying the nonthermal biological effects of THz waves and exploring the physical laws (Fig. 13).

#### Permeability of ion channels

Ion channels, as critical pore proteins being awarded Nobel Prizes 3 times (years 1991, 2003, and 2021), are undoubtedly essential for biological systems. Understanding ion permeation and successfully regulating it is the key to developing new therapeutic strategies for channelopathies or achieving desired neuronal activities. The breakthroughs occurred in 2021 [119–121] when scientists discovered enhanced channel permeation, neuronal signaling, and sensorimotor or learning behaviors. Specifically, by illuminating a 42.55 THz radiation in resonance with the symmetric stretching modes of the -${\text{COO}}^{-}$ key to the voltage-gated calcium channel and altering the hydrogen bond interactions between the ${\text{Ca}}^{2+}$ hydration water and -${\text{COO}}^{-}$, the permeability of ${\text{Ca}}^{2+}$ increased significantly almost 5 times [119]. Although this work was theoretical (later experimentally verified after years of efforts [122]), the contemporaneous experiments [120,121] validated the proposed mechanism. By radiating a 53.7 THz light that resonated with the stretching vibrational mode of the key carbonyl groups in the potassium channel, the ${\text{K}}^{+}$ conductance was exclusively boosted with the ${\text{Na}}^{+}$ current unaffected, as permeation of ${\text{Na}}^{+}$ channel was dominated by the -${\text{COO}}^{-}$ groups instead of -C=O. This led to the inhibition of low-frequency neural electrical activities and enhancement of high-frequency ones [120]. In another experiment, Zhang et al. [121] used fiber optics to transmit light at a close frequency of 53.5 THz directly to the auditory cortex of the mouse brain through the thinned skull surface and found that this frequency-specific stimulation could stably excite cortical neurons and boost associative learning efficiency 50%. These 3 events were milestones of a promising nonthermal, ion channel-based neuromodulation approach with the fundamental mechanism disclosed, that is, the resonance between the THz irradiation and vibrations of key functional groups controlling the permeation of ion channels. Afterward, extensive works studying permeation enhancement or amelioration of prokaryotic, eukaryotic, or modeled ${\text{K}}^{+}$, ${\text{Na}}^{+}$, and ${\text{Ca}}^{2+}$ channels emerged [122–135]. The influence of different radiation parameters was discussed. Most importantly, new physical mechanisms were proposed and discussed. Apart from resonance or coupling between the THz fields with the key functional groups [122,128–135], modulating the stability of channel structure or key residues in the selectivity filter [123,133–135] could also alter the ion currents. Furthermore, the effective irradiation frequencies could be determined based on the vibrational spectra of bound ions [124,125]. Very recently, an increase in ion coherence by THz excitation reaped a significant enhancement of ion conductance [126].

#### Unwinding, unbinding, and unfolding of nucleic acids

THz wave manipulation of DNA and RNA is likely a promising tool in genetic engineering. Recent molecular dynamics (MD) simulations have manifested for the first time that frequency-specific THz radiation in resonance with the in-plane stretching of purines could speed up the DNA unwinding by breaking the hydrogen bonds formed between the base pairs [136]. This was later experimentally validated [137]. Additionally, this capability was further exploited to drive the assembly of DNA origami [138] and enhance the efficiency of polymerase chain reactions (PCRs) [139]. On the other hand, biosafety of the THz stimulation became a concern. Shang et al. [140] found that 0.263 THz high-power irradiation suppressed the genetic expression and undermined movements of the nematodes. It underscored the need for caution in developing THz-based therapeutic strategies while preventing genetic mutations. Furthermore, THz field modulation has been linked to inhibiting migration and glycolysis of tumor cells by decreasing the chromatin accessibility of associate genes, which was attributed to significant suppression of unbinding between the DNA and histone. This work provides a new paradigm for electromagnetic therapies of cancers [141]. In the realm of RNA research, different THz waves have shown the ability to either promote the mechanical unfolding or alter the structure stability of RNA hairpins in different unfolding phases [142]. Overall, successful modulation of the unwinding, unbinding, and unfolding of nucleic acids lies in the alteration of key and basically strongest hydrogen bond affinities between the double strands or between the strands and proteins, arising from resonantly enhanced vibrations of molecular moieties or specific chemical bonds therein.

#### Permeability of tubular and planar water channels

As an indispensable component of life, water, especially confined water in protein pores or interfacial water on the membranes, is of much importance. The THz spectroscopy and MD simulations were used to probe the properties of water [143,144], e.g., the orderliness of interfacial water was characterized based on its stretching vibrations [145]. Zhu et al. [146,147] revealed that 1.39 and 31.5 THz waves could separately induce the transition of confined water in tubular water proteins and monolayer water in planar membrane channels to a super-permeation phase under strength matching and frequency resonance with the hydrogen bond networks within the channels. This ability to control water’s permeation phase could have significant implications for various biological processes involving water behaviors. For example, very recently, suppression of water migration through TRPV1 ion channels has exhibited intriguing feature of pain relief via solvent-mediated cation flux [148]. The impacts of THz fields were also estimated regarding the single-file water transport [149], the surface wettability of an ordered subnanoscale water layer on a solid surface [150], and water permeating across edge-functionalized graphene oxide (GO) membranes [151]. Furthermore, Zhang et al. [152] showcased an ultrahigh-flux water nanopump formed with the asymmetric wettability membrane channels via asymmetric THz absorption. The uniqueness of THz radiation-enhanced permeation of confined water was nonthermal modulation, as the confined water shaped in different channel structures formed distinct hydrogen networks and showed different spectral and absorption characteristics from the bulk water [146,147,150–152]. It follows that the frequency-specific THz wave could precisely target and alter the molecular interactions within each type of confined water or between water and the channel.

#### Affinity between receptors and ligands

Receptor–ligand recognition based on interactions like ionic bonds, hydrogen bonds, van der waals forces, and hydrophobic interactions underpins myriad physiological processes, making receptors the drug targets for therapeutic intervention. To address the severe side effects induced by potent but high-affinity antipsychotic drugs, Li et al. [153] proposed to significantly accelerate drug dissociation by diminishing the hydrogen bonding and π−π stacking forces between the receptor and drug with specific THz irradiation. The underlying mechanism was low-frequency THz field (4.0 THz) resonantly driven switch of ligand conformation instead of the chemical bonds usually targeted. Another exogenous molecule, nicotine, was later proved to dissociate from acetylcholine-binding protein by disrupting the hydrogen bond and cation–π interactions with THz radiation, which showed implications for treating nicotine addiction and related disorders [154]. The THz technology has also been utilized to intervene in the neurotransmission processes [155–160]. Instead of targeting the ligand to alter the dynamics and binding of ligand–receptor complexes [153–156], in [157], the THz energy was resonantly transferred to the glutamate receptor, which enhanced the receptor activity and brought improvement of cognitive functions in conditions like posttraumatic stress disorder (PTSD). Overall, the THz wave-modulated receptor–ligand binding offered new strategies for pharmacotherapy, and the THz responses of receptor–ligand complexes and their binding thermodynamics and kinetics at molecular levels should be the key research points.

#### Protein assembly and enzyme activity

Peptide aggregation, particularly that of amyloid-β (A*β*) peptides, is an essential hallmark for neurodegenerative diseases like Alzheimer’s. Since THz radiation showed superiority of modulating the intermolecular interactions, it rapidly became an alternative to regulate peptide aggregation. It was interesting to find that 3 teams [161–163] simultaneously paid attention to this scenario. Wang et al. [161] demonstrated that 3.1 THz radiation could promote monomer aggregation at early stages but inhibit it at later stages. Peng et al. [162] experimentally validated that 34.88 THz waves could nonthermally disrupt the formation of Aβ fibril by altering the hydrogen bond networks and secondary structures. On the contrary, Chen et al. [163] investigated the opposite effect where 42.55 THz radiation enhanced the structure stability of A*β*42, which reminded us to be cautious to select the operation frequency of electromagnetic radiation. Although the investigation on nonthermal regulation of the enzyme activities is in its infancy, it was delightful to see that researchers developed a nondrug, noninvasive THz regulatory strategy where 33 THz photons effectively inhibited telomerase activity. This resulted in cellular aging, apoptosis, and DNA double-strand breakage, which seriously suppressed the survival of cancer cells [164].

#### Membrane perforation and permeability

Phospholipids form the basis of cell membranes that regulate the exchange of substances between the cell and its environment. The dielectric dispersion and spectral characteristics of phospholipid bilayers were sketched in the THz band [165,166], underscoring the potential of THz waves to manipulate membrane functioning, which might be conducive to drug delivery. For example, after 10 min of exposure to THz radiation at a frequency of 0.3 to 19.5 THz, PC12 cells experienced a temporary increase in membrane permeability and enhanced nanoparticle uptake, which, however, did not cause significant cell death or physiological damage based on a long-term analysis [167]. Additionally, the formation of hydrophilic pores and enlarged permeability of cell membrane perpendicular to 0.4 THz electric fields were witnessed [168], and the radiation parameters behind membrane electroporation and formation of water bridge were further explored [169], where permeability was found highly dependent on the width and intensity of the bipolar pulse trains. Overall, the impact of THz radiation on membrane permeability was deterministic, yet the mechanistic link between THz irradiation and permeability was still not clear. A tenable mechanistic explanation was the directional effect of THz radiation on charged and dipole molecules in the membrane, resulting in structural changes in the phospholipid bilayer and increased curvature of the membrane [170].

#### Cell activities and animal behaviors

Above we have revealed the fundamental mechanisms of THz bioeffects until levels of molecules and chemical bonds. The research studying the laws and effects of THz radiation at the cell and animal levels is much more extensive. For instance, the impact of different radiation conditions (including THz wave frequency, exposure duration and frequency, spot size, and irradiation power) and the objects being illuminated has been largely reported to date among a wealth of works [27,171–173]. Specifically, the biological responses of primary hippocampal neurons to THz radiation were investigated regarding varying power densities [174]. The role in modulating neural signaling was further emphasized where THz radiation was proved capable of promoting synaptic plasticity by activating the nuclear factor κB (NF-κB) pathway [175], enhancing synaptic transmission and cell differentiation in vitro [176], and promoting growth and signaling of neurons [177]. The transcriptome study showed the influence of THz illuminance on cell proliferation and migration [132]. However, despite pioneering discoveries and promising applications in medicine, the depth of research and the revelation of underlying physical mechanisms and laws are still insufficient. A possible reason is that the radiation frequencies adopted mostly range between 0.04 and 3.6 THz, which fall in the low absorption region of the water spectrum resulting from the hydrogen bond stretching vibrations and intermolecular liberational motions. This makes the mechanism obscure as it is challenging to distinguish between the thermal and nonthermal effects. As a consequence, more efforts are needed for a deeper understanding of the bottom mechanisms to effectively harness THz waves for safe neuromodulation [178].

### Development trends and challenges

**Fig. 14.**
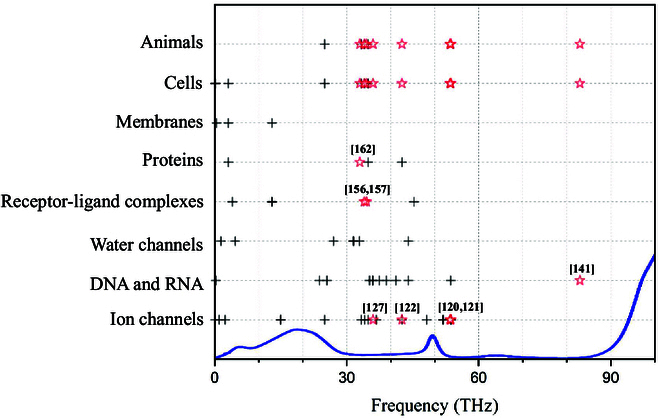
Statistical plot of the theoretical and experimental works on different biological components discussed in this review with respect to the THz radiation frequency. The red stars highlight comprehensive investigations across the animal, cellular, and molecular levels. The experimental spectrum of water absorption [31] is sketched in blue for an easy view of the absorption windows.

Figure 14 summarizes the reported works studying different biological components regarding radiation frequency, which is the most important parameter in THz bioeffects. The research on biological effects induced by high-frequency THz waves (greater than 30 THz) is relatively in-depth, while the one regarding low-frequency THz waves (less than 30 THz) encounters difficulties in determining the bottom molecular mechanisms. This is due to the special infrared absorption spectra of biomolecules. Specifically, the high-frequency band (“characteristic region”) consists of stretching-induced absorption peaks with strong recognizability, easily being used to identify functional groups. The spectrum in the low-frequency band (“fingerprint region”) is complex and somewhat continuous and has low discrimination. While it provides great convenience for bio-sensing in identifying structurally similar compounds, a single low-intensity peak might involve combined vibrations and rotations of collective molecules. Hence, it is difficult to screen the effective radiation frequency and determine the molecular moieties affected. However, the low-frequency THz band is crucial because it mainly involves molecular rotations and molecular conformation changes common in many bioprocesses, such as ion channel opening and closing, receptor–ligand recognition, DNA transcription and translation, protein activation, and enzyme catalysis. In the future, more efforts are demanded to unleash the full capacity of THz technology.

### Perspective

THz biophysics, as an emerging and interdisciplinary field, although developing for 2 decades or so, has already made numerous advancements. Future progress might transform existing paradigms of biological regulation technologies and propel breakthroughs in neuroscience and materials science. To this end, many aspects require our persistent enthusiasm and hard work. First, despite a wealth of THz bioeffects in the nervous system discovered, analyses of the underlying mechanisms demand more effort, particularly in the low-frequency THz region. There is a strong need for a systematic exploration of bioeffects across the entire THz frequency range in the future. Second, although currently there are plausible explanations to the THz bioeffects at the molecular level, the theoretical field strength applied in simulations is at least 4 (or 8 if talking about the power density) orders of magnitude higher than that applied in real experiments. The difference between the irradiation time (e.g., minutes, hours, or days in experiments, while hundreds of nanoseconds in simulations) is a possible factor where longer radiation accumulates more energy. However, we might consider including the possibility of quantum effects that have been verified to exist in many biological processes such as photosynthesis, enzymatic catalysis, and so on, yet are neglected in our classical MD simulations. Although the energy of THz photons is insufficient to cause the formation or breaking of chemical bonds, the THz-based conformational regulation might help lower the reaction energy barriers and increase the occurrence probability of quantum effects like quantum tunneling, thus propelling biological reactions. The challenge is there is no suitable ab initio molecular dynamics (AIMD) approaches dealing with large biosystems together with electromagnetic fields. Also, the computational complexity would be unprecedented. The quantum mechanics/molecular mechanics (QM/MM) method might be an alternative, whereas the oscillating fields should be properly introduced. Last but not least, it is important to study the generation, transmission, and amplification mechanisms of THz information in the nervous system [179], which is the most fundamental problem yet studied rarely [180–182]. Continuously deepening the understanding of THz information characteristics, field transmission media, processes, and methods is expected to break through new physical principles and theoretical frameworks of biophysics.

## Conclusions

Developing advanced detectors—such as SIS mixers, HEBs, TESs, and KIDs—alongside planar Schottky diodes for THz sources, highlights significant strides in astrophysics and THz technology. Efforts to enhance SIS and HEB performance include expanding both RF and IF bandwidths, reducing noise, and increasing array sizes to improve sensitivity, efficiency, and practicality. TES advancements, particularly in microwave multiplexing and QPTAs, simplify readouts and promise future scalability. KIDs are becoming strong contenders in sensitivity and scalability, with research on minimizing TLS noise aiming to enhance their performance. The development of Schottky diodes in THz sources is shifting toward high-power and broadband frequency doubling, driven by new semiconductor materials and circuit designs. The field of THz communication is making progress in increasing transmission rates and distances, focusing on semiconductor-based technologies and optoelectronic hybrids. THz biophysics, an emerging field, aims to explore THz bioeffects comprehensively, advancing understanding across the THz spectrum and unraveling neural information transmission mechanisms. Undoubtedly, the development of high-performance THz sources and effective detectors is the core of the actual practice of THz technologies across the fields of astronomy, telecommunication, and biophysics, in particular regarding the promotion of astronomical detection, THz wireless communication, biodiagnosis, imaging, and effects. In addition, it will advance the interdisciplinary research that involves these 3 fields. A typical case is astrobiology, which relies on THz-based astronomical remote sensing [183] for the detection of organic molecules and the analysis of celestial material to explore the habitable environment of extraterrestrials and life signals [184]. Space exploration [185,186] also serves the field of telecommunications, as the launch and operation of spacecraft require monitoring and diagnosis of the space environment. Apparently, as interdisciplinary research, THz science and technology demand continuous cooperation and contributions of scientists from different disciplines to jointly promote their prosperity.
